# “Did I bring it on myself?” An exploratory study of the beliefs that adolescents referred to mental health services have about the causes of their depression

**DOI:** 10.1007/s00787-016-0868-8

**Published:** 2016-05-20

**Authors:** Nick Midgley, Sally Parkinson, Joshua Holmes, Emily Stapley, Virginia Eatough, Mary Target

**Affiliations:** 1 Anna Freud Centre, 12 Maresfield Gardens, London, NW3 5SU UK; 2 Research Department of Clinical, Educational and Health Psychology, University College London, 1-19 Torrington Place, London, WC1E 7HB UK; 3 Department of Psychological Sciences, Birkbeck College, University of London, Malet Street, London, WC1E 7HX UK

**Keywords:** Adolescence, Depression, Qualitative, Causal beliefs, Understanding

## Abstract

The causal beliefs which adults have regarding their mental health difficulties have been linked to help-seeking behaviour, treatment preferences, and the outcome of therapy; yet, the topic remains a relatively unexplored one in the adolescent literature. This exploratory study aims to explore the causal beliefs regarding depression among a sample of clinically referred adolescents. Seventy seven adolescents, aged between 11 and 17, all diagnosed with moderate to severe depression, were interviewed using a semi-structured interview schedule, at the beginning of their participation in a randomised controlled trial. Data were analysed qualitatively using framework analysis. The study identified three themes related to causal beliefs: (1) bewilderment about why they were depressed; (2) depression as a result of rejection, victimisation, and stress; and (3) something inside is to blame. Although some adolescents struggled to identify the causes of their depression, many identified stressful life experiences as the cause of their current depression. They also tended to emphasise their own negative ways of interpreting those events, and some believed that their depression was caused by something inside them. Adolescents’ causal beliefs are likely to have implications for the way they seek help and engage in treatment, making it important to understand how adolescents understand their difficulties.

## Introduction

It is widely recognised that patients’ causal beliefs about their mental health problems have important implications for how they manage their condition [1] and whether and how they engage with treatment [2]. For example, it has been suggested that patients who take on a biological understanding of their depression—possibly seeing it as something ‘inherited from my dad’ or as ‘a problem with my synapses’—may find pharmacological treatment preferable to psychological therapies [3]. This has been supported by findings that patients who view their depression as chronic are more compliant with medication, whereas medication adherence is poorer in patients who consider their depression to be caused by relationship problems [4, 5]. Less is known about how causal beliefs are associated with treatment preferences and adherence in psychological therapies, but it has been suggested that patients who understand their problems as rooted in childhood difficulties may be more suited to insight-oriented approaches to therapy [6]. To date, research has focused on the causal beliefs of adults regarding their mental health difficulties, but there has been relatively little research in this area focusing on young people.

## Causal beliefs for the onset of depression

When focusing on a clinical population, studies suggest that depressed patients tend to give multi-dimensional explanations for their depression [6]. When asked about their own beliefs, adults with depression have attributed cause to a number of factors. These include traumatic events in childhood and adulthood, stressful life events, inability to cope, personality, and heredity. Few patients considered biological factors to be relevant [3, 7–9], whilst some report a belief that depression is not a ‘real illness’ or that it is a way of medicalizing a normal human experience [10].

Individuals may understand depression differently across the lifespan, which may have important clinical implications for treatment and interventions. One study found that young adults more commonly explained their depression as a result of childhood experiences, middle-aged adults emphasized the role of separation or divorce, and older adults considered loss of a loved one and loneliness to be more common explanations [8].

Adolescents’ causal beliefs about depression have been comparatively under-explored. Hetherington and Stoppard looked at adolescent girls’ understanding of depression, in a sample of high school girls who had not personally experienced depression. These girls considered depression to be the result of a loss of societal connection and social withdrawal; their understanding of the root of depression focused on relational difficulties, such as relationship breakdown and miscommunication with family and friends [11]. Dundon carried out a meta-synthesis of studies exploring the experience of depression in adolescence, covering six qualitative studies published up until 2004 [12]. Of the themes identified across the six studies, two referred specifically to causal beliefs. The first, ‘breaking points’, focused on the way that young people saw the causes and triggers of depression as related to difficult relationships, pressures of being an adolescent, the loss of someone close, and abuse and changes in the family life, such as parental separation. A second theme, ‘seeing and being seen’, described the confusion felt by adolescents who were not able to understand their symptoms, and had no clear set of causal beliefs regarding their depression. This struggle to make sense of the situation was also identified in a more recent qualitative study of the experience of depression in young people [13]. As indicated by a number of studies [14, 15], the nature of the causal beliefs that young people have about their depression is likely to impact the engagement with professional help.

In summary, there is some indication that causal beliefs about depression vary with age, and that adolescents are more likely than adults to attribute their difficulties to psychosocial stressors, such as parental break-up, interpersonal difficulties and experiences of loss. However, to date, studies have been small scale, and have not always been conducted with a clinical sample. Where studies have recruited from mental health services, the young people who participated were already receiving therapy, or had already completed treatment. Therapy is likely to impact on how young people understand their difficulties, as we would expect therapy to lead to some reconsideration of the causal beliefs that the young person brings to treatment [16].

As far as we are aware, there are no prospective studies exploring the causal beliefs regarding the depression of adolescents at the point at which they first receive a referral to a child and adolescent mental health service (CAMHS). The current study aims to address this gap, using an exploratory design, and focusing on the causal beliefs regarding depression among a clinically referred population, prior to starting therapy.

## Methods

### Setting for the study

Adolescents interviewed for this study were taking part in a large, multi-centre, randomised controlled trial, and the IMPACT (Improving Mood through Psychoanalytic and Cognitive-Behavioural Therapy) Study [17]. IMPACT is a pragmatic superiority trial comparing the relative clinical effectiveness of three psychological treatments for adolescent depression, with established evidence of efficacy for evoking clinical remission in the short term (i.e., 3–6 months). The three treatment approaches tested in this study were a manualised form of Specialist Clinical Care termed Brief Psychosocial Intervention (BPI), Short-Term Psychoanalytic Psychotherapy (STPP), and Cognitive Behavioural Therapy (CBT).

### Participants and recruitment

Potential participants in the IMPACT trial were identified by clinical staff from routine referrals to the participating CAMHS clinics. The assessing clinicians informed the young person and their parents/carers about the trial and invited them to consider taking part. The participants and their parents or legal guardians were then sent information sheets about the trial and were asked if they were willing to be contacted by a researcher, who then met participants and invited them to sign a consent form. In agreeing to participate in this pragmatic clinical trial, the young people were agreeing to be randomised to one of the three treatment arms.

A total of 465 young people were recruited to the IMPACT study, with a mean age of 15.6 (SD 1.4). A total of 348 (75 %) were female and 117 male (25 %). 85 % (382/450) described their ethnicity as White British. At the point at which the young people had been recruited to the study, all of them met diagnostic criteria for moderate to severe depression, based on the Kiddie-SADS [18]. The IMPACT study took place across different parts of the UK; the recruitment numbers were 127 for North London, 185 for East Anglia and 153 for the North–West (Manchester and the Wirral).

Alongside the IMPACT study, a sub-study aimed to explore the experience of the participants recruited to the London arm of the IMPACT trial [19]. Recruitment to this sub-study began slightly later than the main clinical trial, in September 2011, at which point the qualitative Expectations of Therapy interview was added to the baseline assessments with adolescents being recruited into the IMPACT trial. All young people recruited in London between September 2011 and December 2012 were interviewed using the Expectations of Therapy interview in their baseline assessment for the IMPACT trial, and comprise the sample for this study. The sample reported in this paper is, therefore, 77 young people who were assessed and eligible for the IMPACT trial. Closely matching the total sample in the IMPACT trial, the average age of these 77 young people was 15.86 years and 71 % were female. Based on a review of all key characteristics, this sub-sample was broadly representative of the participants in both the London arm of the trial and the IMPACT study overall.

### Data collection

The Expectations of Therapy Interview (ETI; [20]) was used for the present study. Young people and their parents/carers were provided with a general information sheet about the study, and before the ETI interview began the young people were told that this interview was about their views on the difficulties that had led them to be referred to Child and Adolescent Mental Health Services (CAMHS). At the start of the interview, the researcher explained that they were interested in hearing about things in the participant’s own words and that there were no right or wrong answers. The semi-structured interview explores three key areas: the difficulties that brought young people into CAMHS; causal beliefs and how they understand their difficulties; and expectations of therapy. In line with guidance on qualitative interviewing, researchers were encouraged to follow the young person’s lead, but follow up with further questions to gain as deep an understanding as possible of their experience. Accordingly, the ETI was carried out before the structured diagnostic measures. The interviews for this sub-study were carried out by post-graduate psychologists, who were given a half-day training session in semi-structured interviewing using the ETI, and were offered feedback on interviewing technique following their initial interviews with young people. Interviews ranged in length from 4 to 37 min, and the mean interview length was 12.19 min (SD = 6.23). All interviews were audio-recorded and transcribed verbatim.

### Data analysis

Given that this was an exploratory study, the data analysis was conducted using the framework analysis (FA; [21]). Sitting within the family of broadly thematic approaches, FA provides a flexible but structured approach to data management and data analysis, which is especially suitable for studies which have focused research questions, a large amount of qualitative data that needs to be managed, and a priori issues to investigate. It also lends itself to studies where qualitative data analysis is carried out by a team working together, rather than individual researchers. The approach has also been integrated with the NVivo 10 qualitative software package [22], which was used in this study. We followed the five stages of framework analysis (familiarization; developing a framework, indexing, charting and mapping and interpretation) [21] to explore patterns in the data and identify the causal beliefs regarding depression expressed by the young people. A reflective account of our experience using the framework analysis has been published elsewhere [23]. The first step, familiarization, involved listening to interviews, reading transcripts, and the whole research team (made up of two clinician-researchers, one academic with expertise in qualitative research methods, two postgraduate research assistants, and one PhD student) discussing a number of the interviews together in a series of data analysis meetings. We then developed the framework, which was used to organise the data set. This was framed around our a priori concerns, but also drew on the preliminary stage of familiarization, to ensure that the framework reflected the young people’s concerns. One of the framework categories was specifically in relation to our interest in adolescents’ causal beliefs about their depression, which was named “understanding of the young person’s difficulties”. The framework was piloted on several interviews, and was revised several times until we felt it provided a good fit with our data.

This framework was then used for the coding process, which involved “indexing” the data (i.e., applying each chunk of text in the interviews to one or more framework categories) and “charting”, where it should be put into chart form, thereby summarising what each interview taught us in relation to each category in the framework, for each participant. The framework was then used to code the 77 interviews used in this study, which meant that whenever a participant spoke about anything in relation to causal beliefs about their depression, it was coded to the “understanding of the young person’s difficulties” category. This would make it easy to extract all information from each interview relevant to our research question in the subsequent “mapping and interpretation” stage. Although most comments about causal beliefs came in one section of the interview, we coded the entire interview for each young person, both to ensure that we included all comments that shed light on their causal beliefs, and also so that we would take other aspects of participants’ experience into account when trying to understand the context for their statements about causal beliefs.

The initial coding process was primarily a data reduction task, to provide a more manageable data set for the later work of interpretation. Three researchers carried out the coding: two postgraduate research assistants (SP and JH) and one PhD student (ES). To ensure that coding was being carried out in the same way, we each coded several of the same interviews and cross-checked our coding. Where discrepancies occurred, they were discussed in team meetings with senior colleagues, to allow us to continually refine the framework and clarify when each of the framework categories should be used.

The final stage was “mapping and interpretation”, which is the analytic phase of framework analysis. Once all of the interviews had been coded to the framework, we had a data summary for each category and participant. Two members of the team (NM and SP, one of whom is a clinician researcher, and the other a postgraduate research assistant) individually explored groups of ten participants’ causal beliefs about their depression at a time, and began to develop a set of themes in relation to adolescents’ causal beliefs about their depression. Having done this individually for each set of ten interviews, they then came together to compare their interpretations. Both brought quite different perspectives to the analysis, with the clinician-researcher drawing on their clinical experience and being more familiar with the theoretical literature on adolescent depression, while the postgraduate research assistant was more in touch with the adolescents’ experiences, being responsible for data collection and having coded many of the interviews. This allowed us to challenge each other’s interpretations and to think about our participants’ experiences from different perspectives, which enabled us to become more confident in our final interpretations. Having developed three core themes regarding causal beliefs based on an analysis of the first 40 interviews, we then tested these themes on our remaining data set. This led to the refinement of our themes, and increased our confidence in the robustness of our thematic structure to encapsulate the views regarding causal beliefs described by the participants in our study. Our final credibility check was sharing our findings with the wider IMPACT-ME team (which included an expert in qualitative research, a clinician researcher, a postgraduate research assistant and PhD student), allowing for a range of different perspectives.

We found that the data fitted well in the three themes that we report below. The biggest challenge that emerged was when adolescents spoke about issues that we considered to be risk factors for depression (e.g., high levels of parental conflict), as it was not always clear whether adolescents necessarily attributed them causally to their depression. Therefore, we opted for caution so as not to impose our own assumptions about adolescents’ causal beliefs about their depression, and excluded these unless the adolescents themselves made links between these factors and their depression. This was where we found it particularly useful to be conducting the analysis as a team, as we were able to challenge each other’s interpretations and go back to the raw data to ensure that we were confident in our interpretations. Where uncertainty around elements of our interpretation remained, we have made this clear in the findings.

### Ethical issues

The study protocol was approved by Cambridgeshire 2 Research Ethics Committee, Addenbrookes Hospital Cambridge, UK (REC Ref: 09/H0308/137). Informed consent was obtained from all participants in the study. Identifiable details were excluded or disguised, and participants chose a pseudonym or if they did not wish to were assigned a pseudonym by the research team.

## Findings

In conducting our analysis, we were able to identify three broad themes concerning the causal beliefs regarding depression among our participants: (1) bewilderment about why they were depressed; (2) depression as a result of rejection, victimisation, and stress; and (3) something inside is to blame. Although we have not aimed to quantify their experiences, to give some indication of frequency, we have used the following system in reporting the findings:

Most of this finding was based on data from 60 or more of the 77 interviews

Many of this finding was based on data from 33 to 59 of the 77 interviews

Some of this finding was based on data from 15 to 37 of the 77 interviews

A few of this finding was based on data from less than 14 of the 77 interviews

1. Bewilderment about why they were depressed: ‘I don’t know, it just happened, it just grew’ (Poppy, 17).
  When asked how they understood their difficulties, many young people struggled to engage with this question. Participants initially responded to questions about how they made sense of what was going on for them by saying that they did not know how things had come to be this way, such as Jake (17), who was quiet for a moment before saying: ‘hmm I am not really that sure really… hmm’. Although expressions such as ‘don’t know’ and ‘not sure’ could be seen as conversational markers, the difficulty in understanding their situation was clearly frustrating for some young people. Jake, for example, described it as ‘really annoying’ not knowing why things were this way. For others, the frustration seemed to come out of a need to justify feeling this way, such as Jenny (15), who said: ‘I just feel like I have nothing to be upset about, which makes me even more upset’.
  While some people seemed to want to understand why things were this way, others conveyed a sense that it was too painful to even think about. For example, Lola (16) said:
  I do not really like to think too far into things I know are gonna make me upset… so I have never really like sat down and gone through all the thoughts and that’s why they have got so jumbled…
  Others described the onset of depression as unexpected. For example, Mina (17) described a bewildering and sudden change one summer:
  I got my [exam] results which were really good and so you know I don’t have much of a reason […] I should have been really happy, I got into the 6th form that I’d wanted to go to, and everything and I started and it was like, it was ok at first….and then like I talked to people, I met new people, but then suddenly I started losing interest, it was quite slow the whole like cutting myself off from everything but it like gradually snowballed, like suddenly I could not stand the company…
  Several young people described that depression had ‘just happened’ (Sofia, 17), and that the onset was not connected to anything specific: ‘I can’t think of a single event or anything that sparked it off’ (Poppy, 17). Others appeared to be attempting to make sense of their situation, but described how difficult this was. For example, Dylan (15) said:
  It’s not really easy to make sense of ‘cause sort of when you’re in that mood, you do not think of anything like you don’t think logically, but then like once you’ve sort of calmed down and everything, I sort of sit and think ‘Why was I like that?’[…] And it doesn’t really make sense.
  For many young people, at the start of interviews, they often had no ideas about why things were this way but as the interview went on, they began to search for meaning and articulate causal beliefs. For example, Ellie (16) initially had no ideas about how she understood her difficulties, but then went on to speak about a range of factors that she saw as connected to her situation:
  The combination of things. I think some pressure and stress from school and just from my sleeping problems have been really bad for the past few weeks, it is just, then all of its combined, and it is just made it worse…
  Some young people spoke about difficulties which are risk factors for depression, but did not connect them to the way they were feeling. For example, a few young people spoke about parental mental health, such as Martin (17) who spoke about how his dad has had ‘serious problems with depression’, but did not specify that this was causally linked to his own difficulties. Other young people spoke about adversities, such as the loss of a parent (Gemma, 15) and bullying (Beth, 16), but did not identify these experiences as causes of their own depression.
2. Depression as the result of rejection, victimisation, and stress: ‘it’s too much pressure on me and it just builds up inside…’ (Nicole, 17).
  Many young people associated the onset of their difficulties with stressful experiences. These stressors were often to do with relational difficulties, including feelings of rejection, victimisation, and loss. Young people spoke about rejection most commonly in relation to family, and especially verbal abuse, which led to them feeling bad about themselves. For example, Nicole (17) spoke about the difficulty of having cared for her mum who had been ill, about how her mum would say to her ‘oh like you’re stupid or ugly’. She described how this affected her:
  I felt sad as well for letting her down […] like I’m not good enough, I do not try hard enough…. So I can not love her enough…
  Judi (17) described how verbal abuse from her aunty ‘made me feel like really down about myself and just doubting myself every time’, whilst Lizzie (17) felt that her mum was not interested in being with her once she had started a second family with a new partner. The sense that the young people conveyed of how it felt to be rejected or unwanted, especially by a parent, were sometimes extremely painful to hear. Aleksander (16), explained:
  My real dad do not get in touch with me at all. Like, it was my birthday and he did not even sent a text or anything to say happy birthday. So I suppose that like, I think that sucks and it makes me feel like crap, if like my Dad doesn’t care, who will? So I suppose that could be why I’m where I am.
  Some young people in our study spoke about having witnessed physical violence at home, which often seemed to be connected to a parent having been inconsistent in their life. Several linked their difficulties with having seen fights between parents. For example, Hayley (17) described how having witnessed her father beating up her mother and siblings was connected to her own difficulties with anger and aggression:
  I thought it was the right thing to do so I had tried to fight with my siblings, but as I grew up I knew it wasn’t the right thing to do, and I just… my anger issues are probably brought from him and what I see him doing…
  Although Hayley links the domestic violence to ‘anger issues’, rather than directly to her depression per se, for many of the young people in our study, their own violent outbursts left them feeling bad about themselves, and in that sense anger and depression were linked. In some cases, a sense of anger, feelings of rejection and loss were all bound together in the way the young people made sense of their difficulties. Megan (14) described how she was ‘so angry’ about her father’s violent death when she was a child. This appeared to have left her with a sense of rejection which she linked to her own depression and anger. For others, the loss that was described might have been related to a relationship, a place or a time in their life.
  For example, Oliver (14) spoke about his life changing as the result of his parents separating, which he considered to be connected to his depression. Eleni (13) also described the changes in her life, as she had lived in a different country, and had moved house and school several times. She described how the upheaval of having moved around had left her feeling:
  Not really safe ‘cause like I feel like as soon as I make friends or I get settled in again that I will just like have to be, I’d have to go somewhere else, or like move somewhere else or somewhere different…
  While many of the young people described one specific cause of their depression, others described a multitude of factors, and it was evident how much pain and adversity many of these young people had been through. For example, Lola (16) described the violence she witnessed between her parents, her father’s inconsistency in her life, and a number of other family difficulties. She went on to describe how this had left her feeling:
  There’s loads and loads of things that are flying around in my head and I can not stop them and look at them and find out what exactly it is and what caused them, I just know that like when it happens it makes you feel sick and dizzy and just horrible…
  Another common factor that young people described when making sense of their situation was to do with relational difficulties with peers. Beth (16) felt that her difficulties with self-esteem started with the bullying she experienced as a child, and when asked why she thought things had become this way, she said ‘it’s because I always feel like I don’t do nothing right in anyone’s eyes’. Similarly, for Brian (12), there was a sense of having given up on trying to make connections with peers because of the hurt they had caused him in the past:
  What’s the point of like even trying to make any friends at all, if they are only ever going to hurt me, or turn their back on me?
  Some young people conveyed a sense of being a victim, such as Gemma (15), who described how ‘it all started from being bullied’. She described how the bullying had started when her dad passed away, which had made her an ‘easy target’. Participants often saw this victimisation as the start of their difficulties, leaving them feeling bad about themselves and withdrawing from their peer group, which left them socially isolated.
  The most common stress described by adolescents was that of school and education—and especially the increased pressure of preparing for major exams, which in the UK usually take place when young people are 16 and 18. Erhan (15), like a number of other young people in the study, described how exams ‘caused me to be depressed’, whereas for some, it seemed to be to do with a more global difficulty with school:
  I think it’s mainly to do with school, I think. Because when I’m at school it’s like it’s dark like there’s nothing to do, nothing to make me feel good or I can not I do not know I just feel moody at school… I feel like school sort of brings me down…
3. Something inside is to blame: ‘I made too big a deal and it’s like a snowball’ (Hakan, 15).
  Although many young people felt that experiences in their lives in some way caused their depression, or at least helped them to understand where their depression came from, some young people saw their difficulties as coming more from within them, or that they were a part of who they were. A few young people offered biological explanations for their difficulties, such as Lizzie (17), who linked her depression to her ‘hormones’, before going on to describe how she had never been truly happy; perhaps suggesting this was just the way she was.
  Some offered a genetic explanation, such as Poppy (17), explaining that other members of her family were also suffering from depression, and suggesting that in some way, this was passed on within the family. Sabrina (17) came up with a range of explanations for why she thought she was depressed, which included considering whether genetics or environmental factors were connected to her situation, but there was a sense of inevitability that things would turn out this way for her:
  I do not know if it’s genetic or if it’s actually physical or if it’s environmental but I am prone to um feel like this and I guess it’s a combination of factors, um I do not know, maybe I feel like this whatever’s going on in my life…
  Others described how they had seen their difficulties as a ‘teenage phase’, such as Ada (17) and Rhianna (15) who would justify her crying to others by saying ‘I’m a teenager… and that’s what teenagers do…’.
  Young people often blamed themselves for their difficulties, such as Kyle (11), who spoke about how things had been this way ‘since I was born […] I have done so many things wrong’, suggesting he saw it as his own fault for the way things had become. This in turn led to a sense of guilt, which exacerbated his depression. Others, although they spoke about external difficulties, such as school and bullying, understood their depression as connected to their response to these adversities, rather than the adversity itself. For these young people, the problem seemed to come from inside them, even if they had experienced significant experiences of victimization or rejection. For example, Lana (14) spoke about how ‘I think I have just let, let kind of stuff get on top of me and I have just let the bullying just get to me…’, suggesting it wasn’t the bullying that led to her depression, but that it was her own fault for allowing the bullying to get to her. Similarly, Hakan (15) spoke about how the stress of exams got too much and there was a sense of self-blame for allowing this to get on top of him:
  Obviously GCSEs and stuff like that, I mean […] I made too big a deal and it’s like a snowball and when you just roll it down the hill it’ll start getting bigger and bigger and that’s what happened to me. I mean everything was getting bigger and I know I should not make it this big but it was just getting bigger and I let that snowball roll down instead of putting it somewhere safe if you know, but that’s life innit. And it just kept rolling down and it kept getting bigger…
  Other young people spoke about their difficulties as being part of their intrinsic personality or character, which again, sometimes represented the way in which young people blamed their difficulties on who they were as a person. For example, Shauna (14) said ‘I don’t think I handle things very well so I think that when things happen to me, I over analyse things so I worry way too much and then I think it’s what I sorta brought it upon myself’. Claire (17) also wondered whether it was because of who she was: ‘maybe I’m just like a really pessimistic person’. Similarly, when asked what would need to happen for things to get better, Danae (16) commented ‘just me, like it’s not really about anything significant that has happened or it’s just kind of me and the way I feel. Like it’s not triggered by anything, I am just the way I am, kind of thing’.

## Discussion

This was an exploratory study, aiming to understand the causal beliefs regarding depression among clinically referred adolescents (aged 11–17). Using qualitative, semi-structured interviews, we hoped to gain an understanding from the perspective of young people themselves.

In line with the previous studies [e.g., 13], our study suggests that young people struggle to make sense of why they feel different from other people their own age, and where their depression has come from. Yet, most of the young people interviewed expressed a wish to understand why they had become depressed, suggesting—as another study found with adults [24]—that giving meaning to one’s experiences may be an important part of creating a sense of order and re-establishing a feeling of identity for depressed adolescents. The previous studies, conducted after mental health service involvement, suggest that adolescents can express ideas about the origins of their difficulties, indicating that such lack of understanding around causation may be a feature of depression, and developing some understanding could be a therapeutic aim it itself.

Our second theme, which described the rejection, victimisation, and stress that adolescents saw as responsible for their depression, fits more closely with the previous findings, including the ‘breaking points’ described in Dundon’s meta-synthesis [12]. Attributing their difficulties to a range of stressful experiences indicates that there is a match between the way young people themselves make sense of their depression and the developmental research, which suggests that a range of environmental factors, including poverty, parental mental health difficulties, peer relation breakdowns, and stressful life events, is indeed risk factors for the development of depression in young people [25]. However, it is striking that the most common form of stress described by the young people in our study was education and exam stress—an area that is relatively under-emphasised in the developmental research literature, which has focused predominantly on events, such as family disruption, divorce, bereavement, and parental rejection or hostility. It may be important for future research that more account is taken of the stress of school and exams, given that the highest incidence of depression in young people coincides with one of the periods of the greatest school pressure in most developed countries.

Our third theme describes how adolescents saw their depression as caused by something inside them, often blaming themselves for feeling this way—even when they also spoke about a range of stressful life events. This supports Beck’s cognitive-diathesis model of depression, which proposes that those who are exposed to stressful events and have negative ways of interpreting them are more vulnerable to depression [26]. In line with the previous research [27], many of the young people in this study demonstrated ‘negative attributional styles’ when speaking of difficult events in their lives, such as bullying or parental hostility; and for some, even their bewilderment about the origins of their depression (theme one) could become a source of self-criticism when they expressed a sense that they should know why they were depressed. Whether such a self-blaming style of thinking is a vulnerability marker or a result of their depression cannot be concluded from this study, but it does indicate the importance for mental health services of addressing the sense of self-criticism and blame that is the characteristic of many young people presenting to services with depression.

## Clinical implications

The present study highlights substantial differences between the young people who took part in this study regarding the causal beliefs they held concerning their own experiences of depression. Although most of the participants in this study struggled to make sense of their depression, they also tried to engage with the issue of why they were depressed, and—perhaps because of the relatively open-ended nature of the interview process—many of them were able to identify some causal beliefs as the interviews progressed. Some young people viewed their depression as specifically caused by difficult experiences, others viewed it as coming from within, as part of who they are—and for many, there was an implicit idea that their depression was the outcome of an interaction between the two.

Such differences may influence how likely adolescents are to seek help for their difficulties, as well as their treatment preferences and how they engage in treatment. For example, young people who see their illness as part of who they are may be particularly at risk of not recognising something is wrong or not seeking support, as they may not believe that their difficulties are something that can be helped. How young people make sense of their condition may also have important clinical implications in guiding clinicians to offer treatments to young people that are relevant and meaningful to how they view their difficulties, or at least to recognize that the treatment may not fit with the young person’s perspective. For example, young people who see their depression as stemming from current stress and pressures in their lives may be particularly suited to an approach, such as cognitive-behavioural therapy [28], which can provide them with strategies to cope with the stressors. Those who think they are stuck in a depression related to earlier events, or who struggle to understand it, may be suited to a more exploratory approach to therapy, such as psychodynamic therapy [29], which may help them to find a framework for earlier events and reactions, and make sense of the difficulties that led them to seek help. Further research is needed to test whether different causal beliefs are associated with engagement in different types of therapy.

Adolescents’ understandings of their depression may also be related to treatment preferences; for example, medication may be more acceptable for the relatively small number of adolescents who see their depression as rooted in biological or genetic explanations. Whatever the explanatory framework held by the young person, it is likely that treatment will be more successful if the clinician is able to explore the young person’s understanding of how they came to be depressed. Given the emerging evidence in adult studies for a link between a shared understanding of the problem and improved clinical engagement and outcomes [30], a case could be made for adding a brief opening module clarifying causal beliefs to all forms of psychological therapy for adolescents suffering from depression, which could help to inform and shape the therapeutic intervention offered. If the young person’s initial view of their depression does not fit a treatment model which is going to be offered (e.g., because of evidence-based protocols), then there might will be a better compliance and response if the clinician acknowledges the discrepancy and explains their reasons. For example, a clinician prescribing medication to a young person who sees their depression as due to recent life events and not an ‘illness’, would do well to give the young person an opportunity to talk through the life events, coping strategies, etc., in addition to explaining the evidence that medication could be effective.

## Limitations

One limitation of our study is that, in using qualitative interviews, there is a risk of our findings being led by the more articulate and open participants, while it is more difficult to hear the voices of those who are less articulate. However, most adolescents in our study were able to engage with the interview and were able to share something about how they understood their difficulties; and in the data analytic process, we were careful to go back to the interviews of the less articulate participants, to ensure that our analysis did justice to their experiences. A comparison group of adults with moderate to severe depression would have also helped to identify which causal beliefs are specific to adolescents, but such a comparison was beyond the scope of the current study. Another limitation is that the study only explored causal beliefs from the participants in the London region of the trial, so it is unknown how representative these findings are of the full IMPACT sample. Although there were no clear differences between the young people in London and other regions, in terms of family background and depression history, it is possible that the different contexts in which they were growing up (e.g., London’s urban environment compared to East Anglia’s larger rural population) might have made a difference in terms of the kind of causal beliefs they held regarding their depression.

## Conclusion

This study highlights the range of understanding in the way young people make sense of their depression. Although some adolescents struggled to identify the causes of their depression, many identified stressful life experiences as the cause of their current depression. They also tended to emphasise their own negative ways of interpreting those events, and some believed that their depression was caused by something inside them. Adolescents’ causal beliefs are likely to have implications for the way they seek help and engage in treatment, suggesting that mental health professionals need to pay careful attention to the way young people understand the difficulties that may have brought them to the attention of services.
